# A Recent Epidemic of Measles at Rotuma

**Published:** 1913-03-08

**Authors:** 


### A Recent Epidemic of Measles at Rotuma.

At the meeting of the Section of Epidemiology, Royal Society of Medicine, on 28th ult., Dr. Hamer in the chair, Mr. Glanvill Corney, I.S.O., gave a very interesting account of a severe epidemic of measles in an island near the Fiji group, Rotumá, in 1911. The area of the island is only fourteen square miles, and the inhabitants number 1,973.

During the period of leave of the medical officer to the island, on January 29, 1911, a steamer, which unfortunately had a case of measles on board, called at the island. Communications took place, and measles became implanted among the people, for the first time in the knowledge of the generation now living, though the late paramount chief remembered that when he was a boy measles ran through the whole island. From the report of the recent outbreak furnished by the present Commissioner, Dr. Hugh MacDonald, it appears that while this epidemic was raging the deaths numbered 489. Death was most busy among young children and among adults of 20 to 25 years of age. Over 45 years the incidence was comparatively slight. Measles accounted for 326 victims, and it was mostly complicated with ileo-colitis, probably of bacillary origin; in some with tubercular disease of lungs, and in a few with yaws, pneumonia, pregnancy, childbirth, miscarriage; while pulmonary tuberculosis accounted for twenty-six deaths. About three months after the epidemic commenced influenza appeared, and persisted in the island for about a month, and when that ceased mumps prevailed. The death-rate was probably much increased by the habit of the patients of standing in the streams to get cool when they felt the fever upon them.

